# Dr Patrick Lionel Grattan Gallwey (MBBS, DPM, FRCPsych)

**DOI:** 10.1192/pb.bp.114.047605

**Published:** 2014-08

**Authors:** Maggie Hilton

**Figure:**
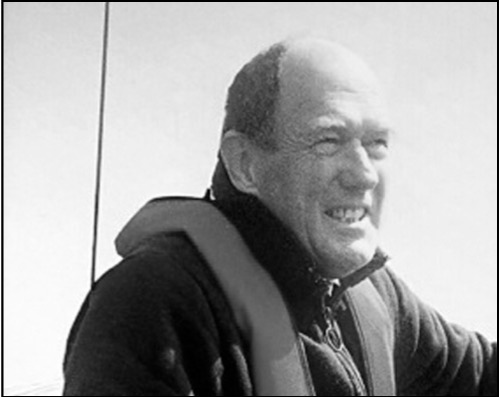


**Formerly Director, South West Thames Regional Forensic Psychiatry Service at St George’s Hospital, London**

Patrick Gallwey, who recently died at the age of 84, was the founding director of the South West Thames Regional Forensic Psychiatry Service. Appointed in 1980, he was the first consultant psychiatrist, who was also trained as a psychoanalyst, in such a role. This dual training, as well as his previous work in HMP Wormwood Scrubs and the Portman Clinic, underpinned the breadth and depth of his understanding of the psychopathology of dangerous people.

Dr Gallwey’s approach to developing a medium secure service was innovative. Rather than establishing a regional secure unit he identified psychiatrists keen to gain experience in this area and used the funds available for a regional unit to pump-prime more local developments. In this way, small secure units were set up in key hospitals throughout the region so that patients would be cared for closer to their homes by teams who would manage their needs for as long as was required. It took courage and determination to get his ideas accepted as many people were understandably concerned about the risks of managing very difficult and potentially dangerous people without considerable physical security. He felt that, in the long term, learning to contain patients psychologically was healthier for them and for staff than relying on physical constraint.

The role of the team at St George’s was to build relationships with staff in these units, attend planning meetings and offer supervision and training opportunities. Seminars were held, rotating through these new services and bringing together staff throughout the region to discuss cases, share problems and to develop their confidence. The most important ingredient was Dr Gallwey’s insight, enthusiasm, tireless energy and belief in the staff’s ability to manage the very difficult issues they were faced with in this work.

He was an original thinker who, by the strength of his humanity, knowledge and charisma, inspired all who worked with him to give their very best and to bring about change. He earned huge respect through his perceptiveness and ability to inspire. When working therapeutically, he managed to tread the difficult path between struggling to understand the unsavoury parts of human functioning while remaining firm and confronting destructive behaviour when necessary.

Dr Gallwey was a great teacher and motivator, conveying to staff his complete trust that they would be able to manage the difficulties inherent in such work. He was always available to advise, reassure and support colleagues grappling with the perverse dynamics typically encountered. His legacy includes many clinicians who, through his teaching, supervision and support, have continued to bring a psychoanalytic perspective to their work, several specialising in psychodynamic psychotherapy and undertaking training in analysis. Other members of staff were forever changed by gaining awareness of the damaging and abusive roots of antisocial behaviour through listening to him giving case illustrations or watching him manage distressed and ‘acting out’ patients. Many of his ex-patients and clients remain able to manage the vicissitudes of life only because of the profound help and support received from him during their troubled pasts. His monthly open clinical seminars at the Institute of Psychiatry were eagerly anticipated by generations of trainees as well as by senior colleagues.

Born in Surrey and evacuated to Devon during the Second World War, he was educated at Dauntsey’s School in Wiltshire. Following national service in the navy, he studied medicine at St Thomas’s Hospital Medical School, University of London. He dropped out for a while to live on a boat and try his hand at being an artist but, after a time, he decided he was not good enough and returned to medicine. After a period in general practice and in junior psychiatric posts, he worked as a medical officer in HMP Wormwood Scrubs providing psychotherapy to some of the most dangerous prisoners. Thus he developed greater understanding of their psychopathology and the impact of therapy in bringing their anxieties to the fore. He also undertook training in Kleinian analysis. Between 1970 and 1979 he was a consultant psychiatrist at the Portman Clinic, working with patients often excluded from other services because of their criminal, sexually harmful and/or violent behaviour.

In 1988, Dr Gallwey left London and moved to Devon to pursue his dream of owning a house in the country with a smallholding. He worked at the Butler Clinic, Langdon Hospital in Dawlish, where he remained until 2001, after which he worked as an independent forensic psychiatric consultant. Throughout his career he saw patients in his psychoanalytic practice and developed an active role as an expert witness. He was on the Editorial Board of the *Journal of Forensic Psychiatry and Psychology* and published papers on psychodynamic processes.

Patrick loved good food and wine, sailing, rugby and the arts, and time spent in his company was always very special, full of humour and wonderful stories. He died on 4 February 2014 and is survived by his much loved wife, Vanessa, and four children, Eli, April, Lucy and Emma.

